# Missing head and color banding in low-count SPECT reconstructions

**DOI:** 10.1186/2197-7364-1-10

**Published:** 2014-09-08

**Authors:** Robin de Nijs, Björn Neumann Jensen, Jann Mortensen

**Affiliations:** 1 Department of Clinical Physiology, Nuclear Medicine and PET, Rigshospitalet, Copenhagen University Hospital, Section 4.01.2, Blegdamsvej 9, Copenhagen, 2100 Denmark; 2 IMT/Biomedical Unit - Section for Image Diagnostics, Rigshospitalet, Copenhagen University Hospital, Section 4.12.2, Blegdamsvej 9, Copenhagen, 2100 Denmark; 3 Faculty of Health and Medical Sciences, Copenhagen University, Blegdamsvej 3B, Copenhagen, 2200 Denmark

**Keywords:** Low counts, Reconstruction, SPECT, ^111^In, Artefact, Missing head, Color banding, Underflow, Integers, Posterization

## Abstract

Due to low counts in an ^111^In single photon emission computed tomography (SPECT) scan, a large part of the head was missing in the reconstructed images on Philips Extended Brilliance Workspace (EBW) and IntelliSpace Portal (ISP) workstations. This problem occurred for the ordered subsets expectation maximization (OSEM) algorithm with and without resolution recovery (Astonish), but not for filtered backprojection (FBP) or maximum likelihood expectation maximization (MLEM). There were also underflow problems because the images are stored as integers resulting in a loss of intensity resolution and color banding.

Philips EBW2.0 and ISP5.02 workstations upscale low-count images, but the result is not always optimal, for example, in the case of low counts in one part and more counts in another part of an image. On these workstations, the missing head artefact problem could be resolved by applying a Hann pre-filter (with a cutoff at the Nyquist frequency, which only influences the filtering) in the reconstruction process. Upscaling of the projection data prior to reconstruction did not recover the head in the images, neither did limiting the reconstructed volume to the low-count part of interest.

Underflow problems were partially solved by the new version 2.0 of the Philips EBW and ISP stations, although situations could arise where underflow still poses a problem. A solution for the underflow problems is to upscale the raw projection data before reconstruction. While this results in a pure upscaling of the FBP reconstruction, the effect in iterative statistical reconstruction is not only upscaling of the intensities because the assumption of Poisson statistics of the data is violated. However, the influence of this last matter seems limited.

Reconstruction of studies with low counts in relevant areas should be performed with care. Reconstruction artefacts and scaling issues can easily arise.

**Electronic supplementary material:**

The online version of this article (doi:10.1186/2197-7364-1-10) contains supplementary material, which is available to authorized users.

## Background

Reconstruction of projection data in single photon emission computed tomography (SPECT) is a complicated process. The corrections for processes arising from physical processes such as attenuation and scatter correction can be achieved by applying several methods. In general, reconstruction can mathematically be performed with analytical and iterative methods [1, 2]. The analytical filtered backprojection (FBP) algorithm is directly derived from the inverse Radon transform but fails to include attenuation and scatter corrections in the reconstruction process. In FBP, the application of a ramp filter can be very noise-sensitive. Iterative reconstruction is a backprojection/forward projection process, where the algorithm seeks to improve the current image estimate with each iteration [3]. In iterative reconstruction, a model of the scanner and acquisition process can be built into the reconstruction itself, describing attenuation, scatter, and limited spatial resolution. The success of this reconstruction depends, to a high degree, on the signal-to-noise ratio (SNR), and therefore, reconstruction of low-count images can be challenging with iterative reconstruction. Iterative reconstruction methods can be classified into algebraic and statistical reconstruction [3]. Depending on the mathematical implementation, the iterative statistical reconstruction is implemented using a maximum likelihood expectation maximization (MLEM) algorithm or an ordered subsets expectation maximization (OSEM) algorithm [4]. The latter is a more efficient version of MLEM. Correction for the distance-dependent spatial resolution can be included in the OSEM reconstruction algorithm and is often referred to as resolution recovery (RR) or point spread function (PSF) correction. Iterative statistical reconstruction assumes Poisson statistics of the projection data and has a non-negative constraint [2].

Images on most scanners and workstations are stored in the Digital Imaging and Communications in Medicine (DICOM) format as 16-bit signed integers. This means that the lowest possible number is -32,768 and the highest number is 32,767. For high-count studies, it is obvious that the images need to be downscaled in order to prevent overflow. This scaling can be stored in the header in the DICOM-field ‘Real World Rescale Slope’. An intercept is also possible. For low-count studies, an underflow problem can arise, when the highest value is low. A highest intensity of 3.4 would mean that there will only be four distinguished colors (0, 1, 2, 3) in the integer format stored image resulting in color banding due to poor intensity quantization. This problem arises from the fact that images are stored as integers and not as floating point numbers. So in the case of low counts, the images also need to be (up)scaled in order to increase the number of distinguishable color levels.

The statistical uncertainty can be so high that reconstruction processes are not capable of assigning counts to the pixels corresponding to the position of the radioactive source. Potentially, this can result in loss of information in larger parts of the image. Also, the underflow problem can result in the disappearance, or non-visualization, of some parts of the image. If the image is stored in integer format, all values between -0.5 and 0.5 are stored as zero resulting in a loss of information. Negative numbers are non-physical but can arise in certain reconstruction methods such as FBP and after filtering.

## Case presentation

A patient with a glioblastoma tumor in the brain was injected with an *ex vivo* preparation of autologous cytotoxic lymphocytes labeled with 10 MBq ^111^In (Autologous Lymphoid Effector Cells Specific Against Tumor cells (ALECSAT) project) and scanned after 24 h on a two-headed Philips Precedence SPECT/16MDCT scanner (Philips Healthcare, Best, the Netherlands) with a 9.5-mm-thick NaI scintillation crystal and medium energy general purpose (MEGP) collimators. Two 20% width energy windows were acquired at 171 and 245 keV and summed consecutively after reconstruction. A low-dose (20 mAs with dose modulation) 140 kVp computed tomography (CT) scan was made of the upper part of the body, and a SPECT scan was acquired at 128 angles (20 s for each angle in step-and-shoot mode) and a 128 × 128 matrix with isotropic 4.664 mm pixels. Total SPECT acquisition time without scanner head movement was approximately 21 min.

**Figure 1:**
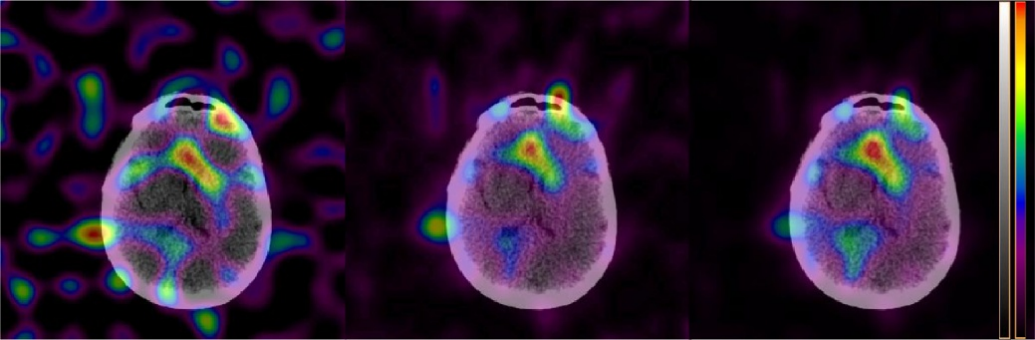
**Transaxial reconstructions fused with a low-dose CT of a slice through the brain with a tumor.** From left to right, FBP, Astonish SPECT reconstruction without attenuation and scatter correction, and Astonish SPECT reconstruction with attenuation and scatter correction (identical to the left panel of Figure 3). Astonish reconstructions are performed with a Hann filter. The left panel shows the noisy FBP reconstruction and can be compared with the less noisy Astonish reconstruction in the middle panel. The influence of attenuation and scatter correction can be observed by comparing the middle and right panel.

**Figure 2:**
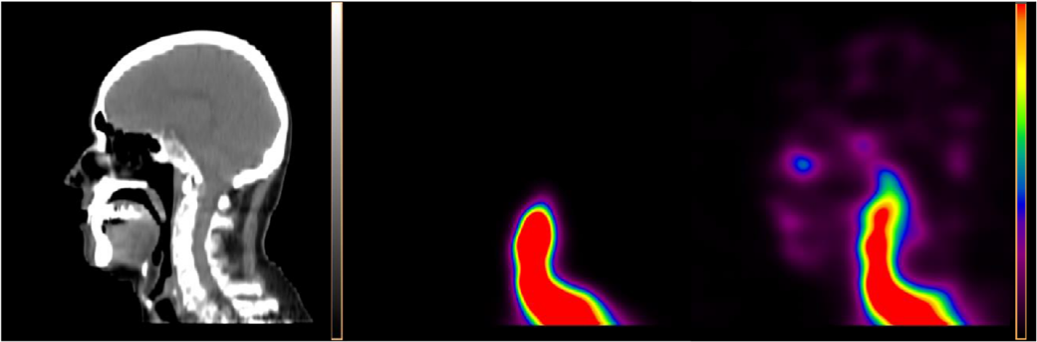
**An example of the missing head artefact shown as sagittal images.** From left to right, low-dose CT scan as an anatomical reference through the middle of the patient's head, Astonish reconstruction, and Astonish reconstruction with a Hann filter. Both Astonish reconstructions are performed with scatter and attenuation correction. The missing head artefact is shown in the middle panel. The Hann filter recovers the head as shown in the right panel.

**Figure 3:**
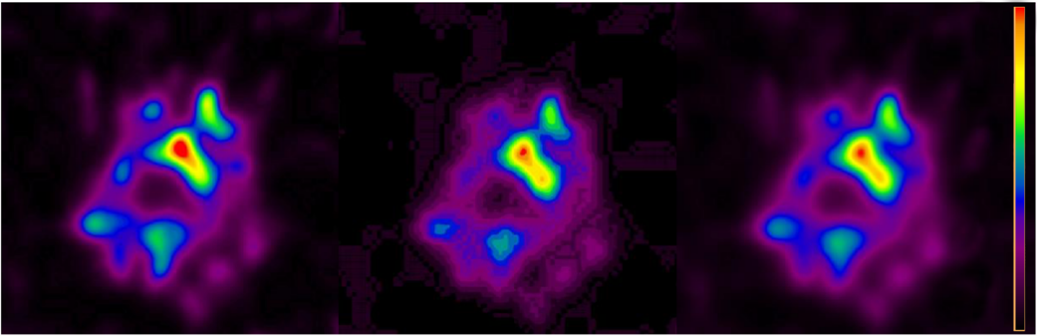
**Comparison of three transaxial Astonish reconstructions with scatter and attenuation correction.** From left to right, Astonish without upscaling, 12× upscaling, and 100× upscaling of the projection data. The color scale is scaled with the same factor as the projection data. Identical slice position as in Figure 1. The images are quite similar, but the middle panel shows color banding, which is not present in the other panels. Upscaling more as in the right panel removes this color banding but introduces minor differences compared to the non-upscaled image in the left panel. The image in the left panel was scaled by the software before storing.

Reconstructions with and without scatter [5] and CT-based attenuation correction [6] were performed with the FBP, MLEM, OSEM, and Astonish method on Philips Extended Brilliance Workspace 4.5.3.40140 NM-version 2.0AB (EBW2.0) and IntelliSpace Portal 5.0.2.20050 (ISP5.02) workstations. Astonish is the Philips implementation of the OSEM reconstruction including resolution recovery. FBP, MLEM, and OSEM were filtered with an eighth order Butterworth filter and a cutoff frequency of 0.25 Nyquist. MLEM was performed with 8 iterations, OSEM with 3 iterations and 16 subsets, and Astonish was performed with 3 iterations and 8 subsets. The Astonish algorithm seems to be best in reconstructing the activity distribution in the brain. For the OSEM reconstructions, the head in the SPECT image disappeared as was the case for the Astonish reconstructions. By applying a Hann [1] pre-filter (with a cutoff at the Nyquist frequency and called ‘Hanning filter’ by the software), this artefact disappeared for the Astonish reconstructions.In Figure 1, a slice of three different SPECT reconstructions fused with a low-dose CT is shown. The maximal pixel value in the raw projection data is five. The noisier FBP reconstruction can be compared to the Astonish reconstruction, as well as the influence of the attenuation and scatter correction for Astonish.Figure 2 shows three sagittal images: an anatomical low-dose CT image and two Astonish reconstructions with scatter and attenuation correction, with and without Hann pre-filter. Without Hann filter, a large part of the image disappeared. This was also the case for OSEM reconstructions. For the OSEM reconstructions, the Hann filter is not available and pre-filtering with the implemented Butterworth filter did not recover the missing head.Figure 3 shows three Astonish reconstructions with attenuation and scatter correction. The left image is automatically scaled by the software (ISP5.02 and EBW2.0 have this implemented, but not EBW1.x). The middle image is reconstructed after upscaling the projection data with a factor of 12, and the right image is reconstructed after upscaling the projection data with a factor of 100. The middle image shows color banding due to underflow problems, which would have been more severe in the left image, if the software had not upscaled the image before storing it.

## Conclusions

Low counts in SPECT can result in poor intensity resolution (color banding) and in the disappearance of some image parts. Color banding can be avoided by upscaling the projection data before reconstruction. Attenuation correction itself can be regarded as a kind of (non-linear) upscaling and can reduce the color banding artefacts visible in the non-attenuation corrected images. Upscaling by the software of the reconstructed data is preferred, since the assumption of Poisson statistics in iterative statistical data construction is not preserved by upscaling the projection data. This may lead to minor differences in the reconstruction as can be seen in Figure 3. Upscaling the projection data or limiting the reconstructed volume to the low-count part of the image did not restore the missing head artefact. Only pre-filtering with the Hann filter removed this artefact.

## Consent

The patient study is part of a clinical trial (protocol EudraCT Number 2011-002180-22, titled ‘Tolerability and efficacy of ALECSAT administered to GBM patients (ALECSAT-GBM)’ that is approved by the Regional Ethical Committee for Copenhagen and conducted in accordance with the Declaration of Helsinki.

Written informed consent was obtained from the patient for publication of this Artefact report. A copy of the written consent is available for review by the Editor-in-Chief of this journal.

## Authors' information

RdN is a medical physicist, BNJ is a biomedical engineer, and JM is a medical doctor.

## Authors’ original submitted files for images

Below are the links to the authors’ original submitted files for images. Authors’ original file for figure 1
Authors’ original file for figure 2
Authors’ original file for figure 3
