# Supplementary figures and images for: Missing head and color banding in low-count SPECT reconstructions
Source: EJNMMI Phys. 2014 Sep 8;1:10. doi: 10.1186/2197-7364-1-10 (PMC6890917; doi:10.1186/2197-7364-1-10)

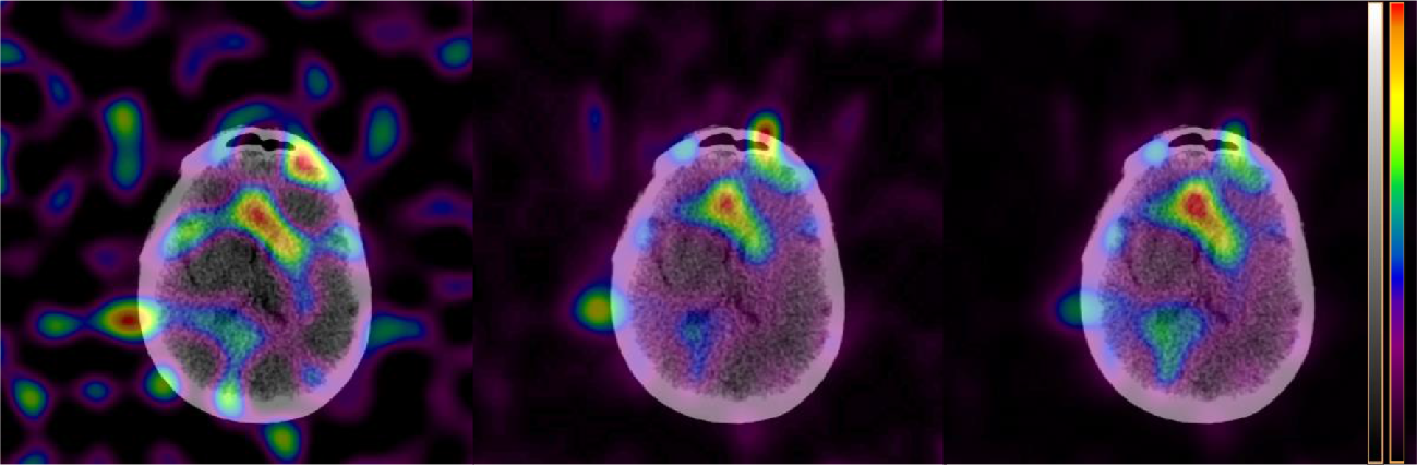

Supplement: Supplementary file 1 — Authors’ original file for figure 1 [file 40658_2014_9001_MOESM1_ESM.tiff]

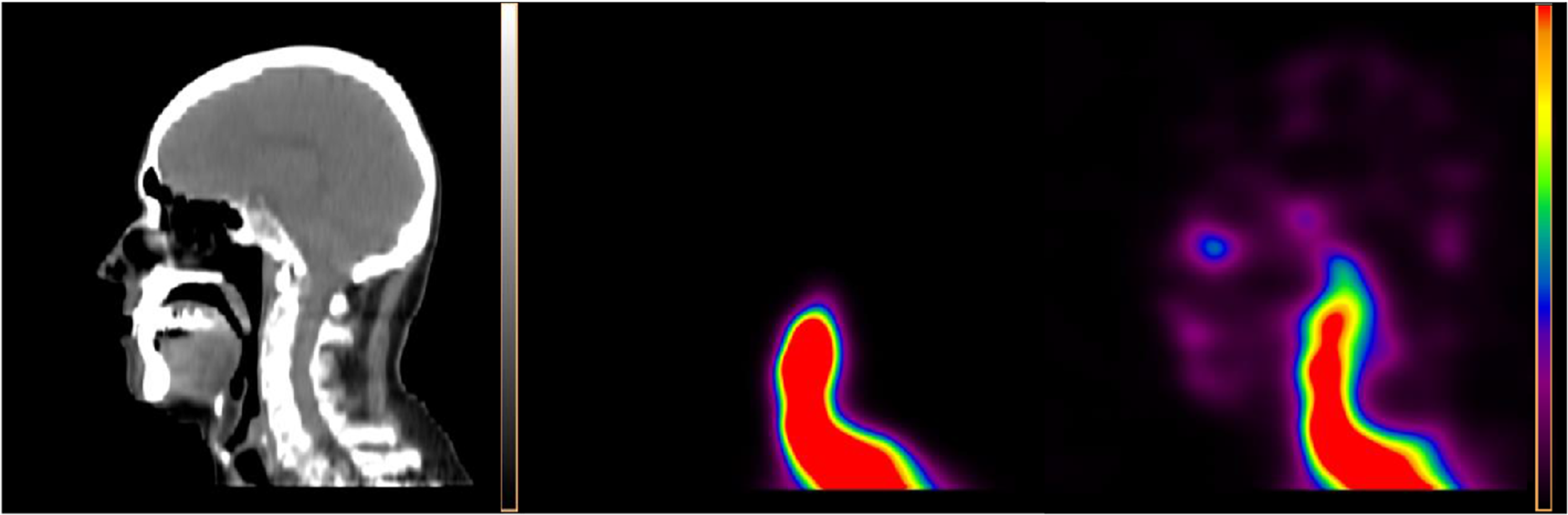

Supplement: Supplementary file 2 — Authors’ original file for figure 2 [file 40658_2014_9001_MOESM2_ESM.tiff]

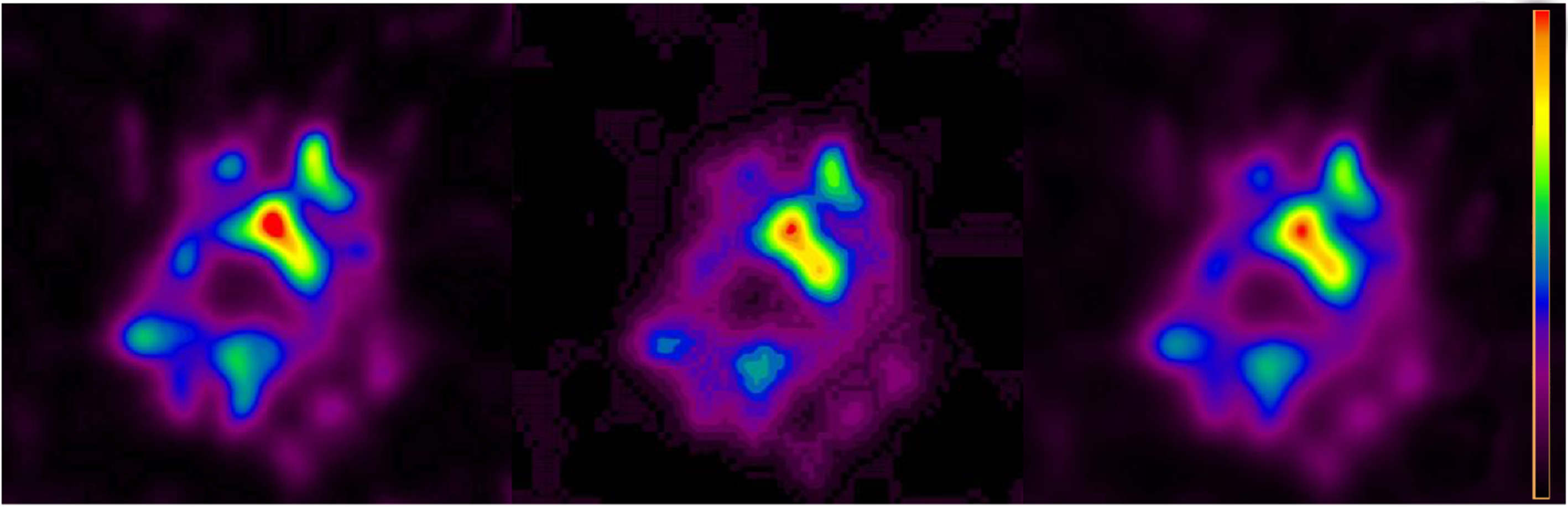

Supplement: Supplementary file 3 — Authors’ original file for figure 3 [file 40658_2014_9001_MOESM3_ESM.tiff]
